# Does Good Aerobic Capacity Attenuate the Effects of Aging on Cardiovascular Risk Factors? Results from a Cross-Sectional Study in a Latino Population

**DOI:** 10.1155/2017/8351635

**Published:** 2017-02-20

**Authors:** Giovanna Valentino, Mónica Acevedo, Lorena Orellana, María José Bustamante, Verónica Kramer, Marcela Adasme, Fernando Baraona, Gastón Chamorro, Jorge Jalil, Carlos Navarrete

**Affiliations:** ^1^División de Enfermedades Cardiovasculares, Escuela de Medicina, Facultad de Medicina, Pontificia Universidad Católica de Chile, Santiago, Chile; ^2^UDA-Ciencias de la Salud, Carrera Nutrición y Dietética, Escuela de Medicina, Pontificia Universidad Católica de Chile, Santiago, Chile; ^3^Departamento de Matemáticas, Facultad de Ciencias, Universidad de La Serena, La Serena, Chile

## Abstract

*Background*. High aerobic capacity is associated with low cardiovascular (CV) risk. The aim of this study was to determine the CV RF burden in subjects with aerobic capacity ≥10 METs and compare it with those having <10 METs. *Methods*. Cross-sectional study in 2646 subjects (mean age 48 ± 12 years). Demographics, medical history, physical activity, cardiovascular RFs, fasting lipids and blood glucose levels, blood pressure, and anthropometric measurements were collected. Aerobic capacity was determined by exercise stress test. The ACC/AHA 2013 pooled cohort equation was used to calculate CV risk. Logistic models were built to determine the probability of having ≥2 RFs versus 0‐1 RF, by age and sex, according to aerobic capacity. *Results*. 15% of subjects had aerobic capacity < 10 METs. The ACC/AHA scores were 15% in men and 6% in women with <10 METs and 5% and 2%, respectively, in those with ≥10 METs. The probability of having ≥2 RFs increased with age in both groups; however, it was significantly higher in subjects with <10 METs (odds ratio [OR]: 2.54; 95% CI: 1.92–3.35). *Conclusions*. Aerobic capacity ≥ 10 METs is associated with a better CV RF profile and lower CV risk score in all age groups, regardless of gender.

## 1. Introduction

The current state of demographic transition in Chile is similar to that seen in developed countries, with a marked increase in the elderly population. In 2015, there were more than 2 million older adults (i.e., those >60 years) in Chile, accounting for approximately 15% of the total population. It is estimated that elderly individuals will constitute approximately 20% of the total population by 2025 and 28% by 2050 [1]. Public health policies that enhance quality of life are critically needed in order to increase the population of healthy older adults, especially since age is an independent risk factor (RF) for chronic noncommunicable diseases, including, most notably, cardiovascular disease (CVD).

According to the last Chilean National Health Survey (NHS 2009), systolic blood pressure (SBP), fasting blood glucose levels, and total cholesterol (total-C) increased by an average of 35 mmHg, 23 mg/dL, and 34 mg/dL, respectively, from the youngest age group (i.e., those 15 to 24 years) to the oldest age group (i.e., those >65 years) [2]. This finding coincides with the high prevalence rates of hypertension (75%), type 2 diabetes (26%), dyslipidemia (42%), and high CV risk (48%) reported for elderly subjects in the same survey [2].

It has been accepted as “normal” that the prevalence of cardiovascular (CV) RFs increases as the population ages. It is also well known that healthy lifestyle habits such as a healthy diet, regular physical activity, and not smoking could have positive effects on CV risk. Several studies have shown a lower risk of total and CVD mortality in subjects with good aerobic capacity, independent of other risk factors, such as obesity and age [3–5]. Despite these findings, most epidemiologic studies are conducted in sedentary populations. For example, in the 2009 Chilean NHS, 89% of the study population were reported to be sedentary—a prevalence rate that increased to 96% in individuals older than 65 years [2]. When analysing epidemiologic data, the association among the different CV RFs, their clustering, and aging in physically active subjects is not well understood.

An aerobic capacity equal to or greater than 10 metabolic equivalents of task (METs; measured during a stress test) has been identified as a good predictor of lower CV risk in medium- and long-term follow-up [3, 4, 6, 7]. However, there is limited evidence in the literature about the CV RF profile in males and females of different ages who have good aerobic capacity. In most longitudinal studies, these subjects are significantly younger, making it difficult to understand how RFs progress in this population [8]. Aging leads to reductions in aerobic capacity as well as increases in both the prevalence rate of CV RFs and the rate of mortality. It is of interest, therefore, to evaluate changes in CV RFs according to age, aerobic capacity, and gender. A recent study conducted in recreational cyclists, aged 55 to 79 and without a history of atherosclerotic or other chronic diseases, showed no significant differences in glucose levels, lipid profiles, and blood pressure (BP) across the age range [9]. Bone density and aerobic capacity were the only parameters that worsened significantly with age. In addition, the investigators reported that there was no association between age and body mass index (BMI) and body composition [9–11]. As this study did not include adults <55 years old, it is unclear what happens with younger, physically active subjects.

The main objective of the present study was to determine the CV RF profiles of men and women with good aerobic capacity (i.e., ≥10 METs) across a broad spectrum of ages and compare them with those of individuals who have low aerobic capacity (i.e., <10 METs).

## 2. Methods

This was a cross-sectional study in 2646 subjects (35% women) who were evaluated in a preventive cardiology prevention program in Santiago between 2002 and 2015. Subjects who were taking lipid-lowering, antihypertensive, and/or glucose-lowering medications and/or those with known atherosclerotic disease and unable to perform a treadmill stress test due to physical disability were excluded. All the subjects signed a written informed consent authorizing the use of their data anonymously for academic purposes.

### 2.1. Data Collection

On the first day, the subjects attended an interview with the nurse coordinator of the program following a 12-hour fast. Demographics, medical history, use of medications, and cardiovascular RFs (including smoking, hypertension, type 2 diabetes, dyslipidemia, and level of physical activity) were recorded in a database. Weight, height, and waist circumference were measured to assess nutritional status according to the World Health Organization and Adult Treatment Panel III criteria, respectively. Blood pressure was measured on 3 occasions on alternate days, and the average was determined as established by the Seventh Report of the Joint National Committee on Prevention, Detection, Evaluation, and Treatment of High Blood Pressure [12]. Finally, venous blood samples were taken to determine lipid and fasting blood glucose levels. The samples were analysed as follows:

1. Total-C, high-density lipoprotein cholesterol (HDL-C), and triglycerides: standard enzymatic methods with ad hoc reactivity (Hitachi analyser)
2. Low-density lipoprotein cholesterol (LDL-C): calculated by the Friedewald formula if triglycerides <400 mg/dL
3. Glucose: glucose oxidase method

The 2013 ACC/AHA pooled cohort equation was used to calculate the subjects' CV risk score at 10 years.

### 2.2. Aerobic Capacity and Level of Physical Activity

Aerobic capacity, indicated as METs, was determined by a treadmill stress test performed until the subject reached the theoretical maximal heart rate or until the subject was exhausted. Subjects who performed ≥10 METs in the stress test were considered to have a “high aerobic capacity,” whereas those with <10 METs were considered to have a “poor aerobic capacity.” It is important to highlight that the phrase “poor aerobic capacity” is used to name the reference group, but it does not mean that the group as a whole had a poor physical condition. Instead, it emphasizes that the ≥10 METs group was indeed a very well-fit group. Finally, we also included the self-reported leisure time physical activity described by the 2009 Chilean NHS to classify the population into three groups according to their reported weekly physical activity frequency: (a) <1 time/week, (b) 1‐2 times/week, and (c) ≥3 times/week.

### 2.3. Statistical Analysis

The sample was divided by gender and age (<55, 55–64, and ≥65 years old) to compare biochemical parameters and BP according to aerobic capacity (≥10 METs and <10 METs). Independent *t*-test samples were used to compare means, and significance was considered a *p* value <0.05.

Finally, logistic models were built to determine the probability of having ≥2 RFs versus 0‐1 RF according to age and aerobic capacity in both men and women. The same analysis was repeated according to the level of physical activity.

The following criteria were considered for de novo cardiovascular RFs (we excluded patients who had pharmacologically treated RFs):

- HDL cholesterol <40 mg/dL in men and <50 mg/dL in women
- Non-HDL cholesterol ≥160 mg/dL
- SBP ≥140 and/or DBP ≥90 mmHg
- Glucose ≥100 mg/dL
- Waist >90 cm in men and >80 cm in women

## 3. Results

Table 1 summarizes the demographic data of the population. The mean age was 48 ± 12 years; the CV risk factors prevalence “de novo” was 10% hypertension, 2% diabetes, 72% dyslipidemia, and 27% metabolic syndrome. Seventy-two percent of the subjects were <55 years old, 20% were between 55 and 64 years old, and 8% were ≥65 years old. Fifteen percent of the subjects were classified as having poor aerobic capacity (<10 METs); these patients tended to be older and more predominantly female. Of those patients ≥65 years old, 37% had high aerobic capacity (≥10 METs). The ACC/AHA 10-year risk score was 15% in men and 6% in women with <10 METs and 5% and 2%, respectively, for men and women with ≥10 METs.

Tables 2, 3, and 4 show the biochemical parameters and BP by age group, gender, and aerobic capacity (<10 METs and ≥10 METs). Patients in the two younger age groups (<55 and 55 to 64 years old) and with high aerobic capacity had significantly lower SBP, DBP, fasting glucose levels, waist circumference, BMI, and ACC/AHA risk scores, regardless of gender. In women ≥65 years old, those with high aerobic capacity had significantly lower fasting glucose levels, waist circumference, and BMI than those with poor aerobic capacity.

**Figure 1:**
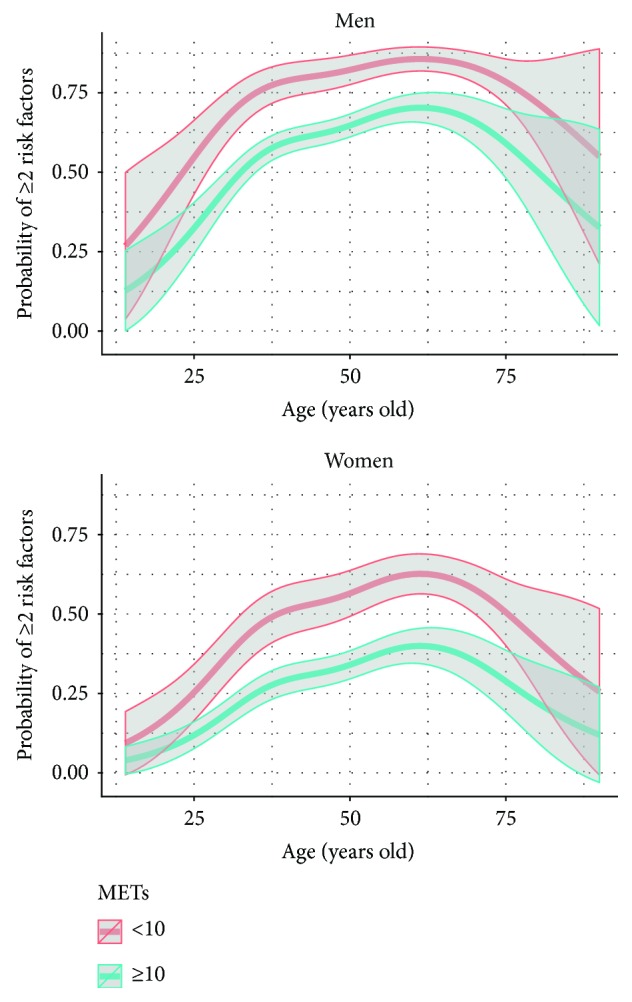
Odds proportional models showing the probability of presenting ≥2 risk factors according to aerobic capacity and age in men and women.

**Figure 2:**
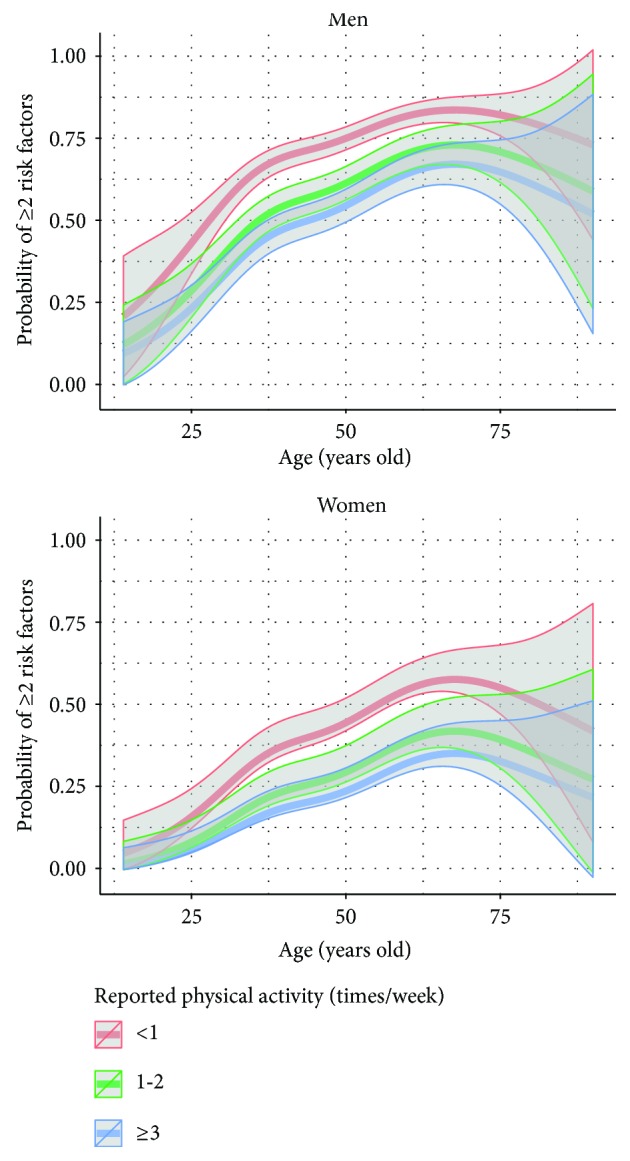
Odds proportional models showing the probability of presenting ≥2 risk factors according to self-reported physical activity and age in men and women.

As shown in Figure 1, the probability (odds ratio) of presenting with ≥2 RFs increased with age in both groups of aerobic capacity. However, the probability of having a higher RF burden was significantly increased in men and women with poor aerobic capacity compared to those with high aerobic capacity (OR: 2.54; 95% CI: 1.92–3.35). When analysed by self-reported levels of physical activity, the results were similar (Figure 2): those subjects who reported physical activity <1 time/week were more likely to have ≥2 RFs when compared to those who exercised ≥3 times/week (OR: 2.51; 95% CI: 2.06–3.05).

## 4. Discussion

This study evaluated the association between aerobic capacity and clustering of CV RFs by age and sex. We have demonstrated that the effect of aging on CV RFs might be attenuated by an individual's aerobic capacity or physical activity level. These results reinforce the concept that fitness level is a powerful predictor of CVD risk independent of age and gender.

Faselis et al. reported similar results in 2012 in a group of 2303 prehypertensive men. In that study, men with an aerobic capacity ≤8.5 METs had 66% higher risk of presenting with hypertension at the 8-year follow-up than those with an aerobic capacity >10 METs [6]. Not surprisingly, the group with the highest aerobic capacity was significantly younger than those subjects with low aerobic capacity (mean age 45 compared with ≥55 years, resp.). It is important to note that Faselis and colleagues analysed their study population based only on aerobic capacity and not by age ranges, as was done in the present study. In another report from this group (published in 2014), there was an 11% reduction in the risk of mortality for every 1-MET increase in aerobic capacity in 2153 hypertensive men who were ≥70 years old [3]. The results from this report suggest that aerobic capacity is a powerful predictor of mortality, likely at all ages [3].

Few studies have reported the association between aerobic capacity and CVD risk in women. Our findings show that, in both adult men and women aged 18 to 90 years, an aerobic capacity <10 METs is associated with an increased risk of having ≥2 CV RFs.

Overall, our study demonstrated that CVD risk is lowest in those individuals with high aerobic capacity (≥10 METs), by age or gender. Of note, however, in male subjects ≥65 years old, only the ACC/AHA risk score was significantly lower in those with good aerobic capacity. These findings could be influenced by smaller numbers of subjects in this age group (116 men and 93 women). When subjects receiving therapies for hypercholesterolemia, hypertension, and/or type 2 diabetes were included (a separate, parallel analysis), SBP, blood glucose, and waist circumference remained significantly lower in elderly men and women with good aerobic capacity (data not shown). Finally, these results were extremely reproducible across the different age groups and when analysing by level of physical activity.

Some studies have previously demonstrated that poor cardiorespiratory fitness has detrimental consequences on CV risk. This has been mainly demonstrated in adolescents and adults. In 2007, Anderssen and colleagues reported that school children aged 9 to 15 with low aerobic capacity had the highest clustering of CVD risk factors (total-C : HDL-C ratio, high plasma triglycerides, sum of 4 skin folds, and SBP) [13]. Another study showed that increased cardiorespiratory fitness (>13 METs) in adult men with the metabolic syndrome was associated with beneficial effects on inflammation [14]. This report also demonstrated that exercise improved insulin resistance, dyslipidemia, hypertension, and platelet function in this cohort [14]. Similar findings were reported by Mora and colleagues in 2007: regular physical activity in a group of 27,055 healthy women was associated with moderate reductions in C-reactive protein, soluble intracellular adhesion molecule-1, and vascular adhesion molecules [15]. However, the investigators stated that the mechanisms underlying the chronic anti-inflammatory and hemostatic effects of exercise were not well defined [15]. Potential explanations for the beneficial effects of increased aerobic capacity include positive effects on proatherogenic adipokines, insulin-sensitizing pathways, or the hemostatic and antioxidant functions of the coronary endothelium [15]. More recently, a study in 781 men and 890 women has shown that both physical activity assessed by a self-reported questionnaire and cardiovascular fitness measured in an ergometer are independently associated with a lower cardiovascular risk [16]. Data from this study were not reported by age and/or sex. Recent studies suggest that the positive effects of exercise might be mediated by an improvement in the biologic age, measured by telomere length [17, 18].

The findings from our study relate to the process of aging. It is undeniable that age is the most important CVD risk factor for CVD and coronary heart disease. All of the CVD risk factors increase with age. However, as shown here, in both men and women who are very fit, from age 55 onward, CV RF burden is less than that seen in subjects who are less well fit. From the public health perspective, these data should be shared at the population level. Public policy designed to educate about the attenuation of the effect of aging on CV RFs by aerobic capacity might encourage the general population to become more physically active. Such efforts could reduce health-related costs associated with an aging population.

Our findings for the younger population were equally impactful. In both men and women, those who had either aerobic capacity ≥10 METs or self-reported physical activity ≥3 times/week had a significantly lower probability of having ≥2 or more CV RFs. This observation is particularly important, given the epidemic of obesity and the metabolic syndrome in Latin American youths. For example, in Chile, the latest results of the National Evaluation in Exercise, performed in adolescents (13–15 years of age), have shown that only 7% of 8th graders had “outstanding aerobic capacity” (defined as >11.5 and >13 METs for girls and boys, resp.), whereas 84% of girls and 55% of boys had an aerobic capacity under the acceptable threshold according to the Navette test (≤10.5 and ≤11 METs for girls and boys, resp.) [19]. These data, published in 2014, were worse than the earlier results from 2010 to 2013, suggesting a dangerous trend. Future generations need to be urgently educated about the importance of the impact of physical activity on cardiovascular risk. Public policies focusing on initiatives that encourage physical activity from an early age are warranted.

This study has some limitations. It is a cross-sectional study, and as such it does not prove causality, and no data on cardiovascular events and/or mortality are reported. The population included in this study belongs to high and medium socioeconomic levels. We measured aerobic capacity using a maximal stress treadmill test, not maximal oxygen uptake. However, all the stress tests were performed by the same team who demanded the patients to make the greatest effort during the exercise in order to really achieve maximal aerobic capacity. Finally, we decided the cut point of 10 METs because most literature evidence underlines that an aerobic fitness of 10 METs is associated with good cardiovascular prognosis, even though it is known that in the extreme groups of age this number could be greater or lower if the median is applied, respectively.

In conclusion, this study demonstrates that an aerobic capacity ≥10 METs is associated with a better CV RF profile and lower CV risk score in all age groups, regardless of gender. Larger studies in active subjects are needed to further elucidate the beneficial effects of exercise.

## Figures and Tables

**Table 1:** Demographics and characteristics of the study population.

Characteristic	Total (*n* = 2646)	Men (*n* = 1776)	Women (*n* = 870)	*p* value
Age, years	48 ± 12	46 ± 12	51 ± 12	<0.0001
BMI, kg/m^2^	26.7 ± 4	27.4 ± 3	25.2 ± 4	<0.0001
Waist circumference, cm	91 ± 12	96 ± 10	81 ± 11	<0.0001
*Biochemical variables*				
SBP, mmHg	120 ± 13	122 ± 12	116 ± 13	<0.0001
DBP, mmHg	75 ± 8	76 ± 8	72 ± 7	<0.0001
Blood glucose, mg/dL	91 ± 14	93 ± 15	86 ± 8	<0.0001
Triglycerides, mg/dL	135 ± 94	150 ± 103	103 ± 60	<0.0001
HDL-C, mg/dL	53 ± 15	48 ± 12	63 ± 15	<0.0001
LDL-C, mg/dL	129 ± 35	130 ± 34	127 ± 35	NS
Non-HDL-C, mg/dL	156 ± 41	160 ± 40	147 ± 39	<0.0001
Aerobic capacity, METs	11.6 ± 3	12.5 ± 3	10 ± 3	<0.0001
Smokers, %	22%	22%	23%	NS
Overweight and obesity, %	65%	75%	45%	<0.0001
Dyslipidemia, %	67%	72%	58%	<0.0001
Hypertension, %	8%	10%	5%	<0.01
Diabetes, %	1.5%	2%	0%	0.02
Metabolic syndrome, %	22%	27%	12%	<0.0001
*ACC/AHA risk score, %*	4.8%	5.5%	3.2%	<0.001

BMI, body mass index; DBP, diastolic blood pressure; HDL-C, high-density lipoprotein cholesterol; LDL-C, low-density lipoprotein cholesterol; METs, metabolic equivalents of task; NS, not significant; SBP, systolic blood pressure; SD, standard deviation. Data are presented as mean ± SD except otherwise indicated.

**Table 2:** Biochemical and anthropometrical variables according to aerobic capacity in subjects <55 years old.

Variable	Men			Women		
	<10 METs (*n* = 51)	≥10 METs (*n* = 1337)	*p* value	<10 METs (*n* = 72)	≥10 METs (*n* = 451)	*p* value
Non-HDL-C, mg/dL	173 ± 40	159 ± 42	0.01	151 ± 36	139 ± 39	0.01
HDL-C, mg/dL	43 ± 11	48 ± 12	<0.01	61 ± 16	62 ± 15	NS
SBP, mmHg	131 ± 14	119 ± 10	<0.0001	118 ± 12	110 ± 10	<0.0001
DBP, mmHg	83 ± 8	75 ± 7	<0.0001	75 ± 7	70 ± 7	<0.0001
Blood glucose, mg/dL	105 ± 41	91 ± 13	0.02	88 ± 7	85 ± 7	<0.001
Waist circumference, cm	107 ± 14	95 ± 10	<0.0001	89 ± 14	79 ± 10	<0.0001
BMI, kg/m^2^	32 ± 5	27 ± 3	<0.0001	28 ± 7	24 ± 4	<0.0001
ACC/AHA score, %	6 ± 4	3 ± 3	<0.0001	1.6 ± 1.6	1.3 ± 2	0.08
≥2 risk factors, %	92%	58%	<0.0001	55%	29%	<0.0001

BMI, body mass index; DBP, diastolic blood pressure; HDL-C, high-density lipoprotein cholesterol; METs, metabolic equivalents of task; NS, not significant; SBP, systolic blood pressure; SD, standard deviation. Data are presented as mean ± SD.

**Table 3:** Biochemical and anthropometrical variables according to aerobic capacity in subjects between 55 and 64 years old.

Variable	Men			Women		
	<10 METs (*n* = 51)	≥10 METs (*n* = 221)	*p* value	<10 METs (*n* = 99)	≥10 METs (*n* = 155)	*p* value
Non-HDL-C, mg/dL	164 ± 35	165 ± 36	NS	160 ± 39	156 ± 39	NS
HDL-C, mg/dL	48 ± 13	49 ± 11	NS	62 ± 16	65 ± 17	NS
SBP, mmHg	134 ± 14	126 ± 12	<0.001	123 ± 12	118 ± 12	<0.001
DBP, mmHg	83 ± 10	78 ± 7	<0.01	76 ± 6	73 ± 7	<0.01
Blood glucose, mg/dL	104 ± 14	94 ± 10	<0.01	89 ± 8	88 ± 9	NS
Waist circumference, cm	102 ± 10	97 ± 8	<0.001	88 ± 11	80 ± 9	<0.0001
BMI, kg/m^2^	29 ± 4	27 ± 3	<0.01	28 ± 4	25 ± 3	<0.0001
ACC/AHA score, %	13 ± 6	10 ± 4	<0.01	4 ± 2	3.5 ± 2	0.06
≥2 risk factors, %	84%	69%	0.04	61%	42%	<0.01

BMI, body mass index; DBP, diastolic blood pressure; HDL-C, high-density lipoprotein cholesterol; METs, metabolic equivalents of task; NS, not significant; SBP, systolic blood pressure; SD, standard deviation. Data are presented as mean ± SD.

**Table 4:** Biochemical and anthropometrical variables according to aerobic capacity in subjects 65 years and older.

Variable	Men			Women		
	<10 METs (*n* = 61)	≥10 METs (*n* = 55)	*p* value	<10 METs (*n* = 70)	≥10 METs (*n* = 23)	*p* value
Non-HDL-C, mg/dL	144 ± 31	154 ± 39	NS	161 ± 41	156 ± 36	NS
HDL-C, mg/dL	51 ± 13	52 ± 13	NS	64 ± 14	66 ± 10	NS
SBP, mmHg	136 ± 15	131 ± 14	0.07	128 ± 13	127 ± 13	NS
DBP, mmHg	79 ± 7	79 ± 8	NS	77 ± 8	75 ± 6	NS
Blood glucose, mg/dL	100 ± 17	98 ± 15	NS	90 ± 10	86 ± 7	0.04
Waist circumference, cm	98 ± 10	97 ± 8	NS	85 ± 13	78 ± 10	<0.01
BMI, kg/m^2^	27 ± 4	27 ± 3	NS	26 ± 5	24 ± 3	<0.01
ACC/AHA score, %	25 ± 10	20 ± 7	<0.001	13 ± 9	9 ± 6	0.02
≥2 risk factors, %	71%	71%	NS	58%	35%	0.06

BMI, body mass index; DBP, diastolic blood pressure; HDL-C, high-density lipoprotein cholesterol; METs, metabolic equivalents of task; NS, not significant; SBP, systolic blood pressure; SD, standard deviation. Data are presented as mean ± SD.
